# Einfluss neurologischer Erkrankungen auf Beweglichkeit und Fahreignung

**DOI:** 10.1007/s00103-024-03920-7

**Published:** 2024-07-16

**Authors:** Günther Thayssen, Klaus Püschel

**Affiliations:** 1 Klinik und Poliklinik für Neurologie, Universitätsklinikum Hamburg-Eppendorf, Martinistr. 52, 20246 Hamburg, Deutschland; 2 Institut für Rechtsmedizin, Universitätsklinik Hamburg-Eppendorf, Hamburg, Deutschland

**Keywords:** Alter, Mobilität, Gangstörungen, Fahreignung, Neurologie, Age, Mobility, Gait disorders, Ability to drive, Neurology

## Abstract

Die Neurologie beschäftigt sich mit organischen Erkrankungen der Muskulatur, der peripheren Nerven von Rumpf und Extremitäten sowie den Erkrankungen des zentralen Nervensystems (Rückenmark, Hirnstamm, Kleinhirn sowie Großhirn). Krankheiten, die Funktionsstörungen dieser Strukturen verursachen, können Beschwerden sowohl körperlicher als auch kognitiver Art hervorrufen. Damit können neurologische Erkrankungen in besonderer Weise die persönliche Mobilität sowohl durch körperliche Einschränkungen als auch durch kognitive Defizite beeinträchtigen. Viele dieser Erkrankungen zeigen im Alter eine deutliche Zunahme der Häufigkeit.

Körperlich bedingte Einschränkungen der Mobilität manifestieren sich schwerpunktmäßig in Form von Gangstörungen. Diese werden in relevanter Ausprägung bei 2 Dritteln der über 80-Jährigen festgestellt und bilden eine häufige Ursache für Stürze mit oft erheblichen Folgeschäden. Beim Fahren eines Kraftfahrzeugs resultieren u. U. negative Auswirkungen z. B. auf Reaktionsgeschwindigkeit, Bremskraft und Schulterblick. Hierfür können die Parkinson-Krankheit sowie Lähmungen und Sensibilitätsstörungen im Rahmen von Polyneuropathien verantwortlich sein.

Das Autofahren ist ein naheliegender Kompensationsmechanismus im Falle von Beeinträchtigungen der Gehfähigkeit. Allerdings liegt bei zahlreichen die Gehfähigkeit beeinträchtigenden Erkrankungen die Ursache im zentralen Nervensystem, oft im Bereich des Großhirns. Folglich können sich neben körperlichen auch kognitive Defizite manifestieren, die einen Verlust der Fahreignung begründen. Als alterstypische neurologische Erkrankungen, die in dieser Weise die Mobilität einschränken, sind hier wiederum die Parkinson-Krankheit sowie Durchblutungsstörungen des Gehirns zu erwähnen. Zudem treten im Alter vermehrt epileptische Anfälle als Symptom anderer Erkrankungen auf.

## Abstract (en)

Neurology deals with organic diseases of the muscles, the peripheral nerves of the trunk and extremities, and the central nervous system (spinal cord, brain, stem, cerebellum, and cerebrum). Diseases that lead to dysfunction of these structures can cause both physical and cognitive problems. Therefore, neurological diseases can particularly impair personal mobility through both physical limitations and cognitive deficits. Many of the diseases show a significant increase in frequency with age.

Physical impairments in mobility primarily manifest as gait disorders. These are found to a relevant extent in two-thirds of people older than 80 years of age and are a common cause of falls, often with considerable sequelae. Driving a car can have negative effects, for example, on reaction speed, braking power, and looking over the shoulder. Parkinson’s disease as well as paralysis and sensory disorders in the context of polyneuropathies can be responsible for this.

Driving a car is an obvious compensatory mechanism with respect to impaired walking ability. However, the cause of many diseases that affect the fitness to walk lies in the central nervous system, often in the area of the cerebrum. Consequently, cognitive deficits manifest themselves in addition to physical ones, which further restrict mobility through the loss of the fitness to drive. Neurological diseases typical of old age that limit mobility in this way include Parkinson’s disease and circulatory disorders of the brain. In addition, epileptic seizures occur more frequently in old age as a symptom of other diseases.

## Einleitung

Die persönliche Mobilität kann durch verschiedene krankheitsbedingte Funktionsstörungen beeinträchtigt werden, von denen meist ältere Menschen betroffen sind. Auf dem Gebiet neurologischer Erkrankungen sind dabei zum einen körperliche Defizite zu nennen, die beispielsweise die Gehfähigkeit und -sicherheit beeinträchtigen. Zum anderen können zusätzlich kognitive Beeinträchtigungen auftreten, die die Fähigkeit zum Führen von Kraftfahrzeugen infrage stellen und die Überprüfung der Fahreignung notwendig machen. Wenn das Autofahren beendet werden muss, kann eine wesentliche Möglichkeit der Kompensation z. B. von Gehbehinderungen verlorengehen.

Das Ziel der folgenden Ausführungen ist es, die Auswirkungen neurologischer Funktionsausfälle auf die Geh- und Stehfähigkeit sowie die Fahreignung darzustellen. Parallel sollen die übergeordneten Aspekte der Kognition und Koordination herausgearbeitet werden.

## Eingeschränkte Mobilität durch Gang- und Standstörungen

Beeinträchtigungen der Mobilität durch Verschlechterung der Gehfähigkeit gehören zu den häufigsten Symptomen einer Behinderung des älteren Menschen. Im Rahmen einer Studie an einer norditalienischen Population („Bruneck-Studie“) konnte gezeigt werden, dass etwa 2 Drittel der Bevölkerung jenseits des 80. Lebensjahres an funktionell relevanten Beeinträchtigungen der Gehfähigkeit leiden [1]. Dabei stellen neurologische Erkrankungen ursächlich die größte Gruppe dar. Mit zunehmendem Alter nimmt dabei der Anteil einer Kombination verschiedener Faktoren im Sinne einer „multifaktoriellen Gangstörung“ deutlich zu [2].

Alle Funktionsbereiche des zentralen und peripheren Nervensystems können ursächlich für neurologisch bedingte Beeinträchtigungen der Gehfähigkeit sein. Dabei können die motorischen, die sensiblen und die koordinativen Systeme, aber auch das Sensorium und die Kognition betroffen sein. Aus dem Bereich nichtneurologischer Erkrankungen mit besonderer Beeinträchtigung der Mobilität sind orthopädische Erkrankungen des Skelettsystems besonders durch degenerative Veränderungen zu nennen. Auch internistische Erkrankungen mit daraus resultierender Einschränkung der allgemeinen Belastbarkeit wie Herzinsuffizienz oder Adipositas kommen als alleinige oder auch zusätzliche Ursachen für eine Einschränkung der Mobilität infrage. Nach einer älteren Studie [3] gehören Gangstörungen zu den ärztlich weniger beachteten und unterdiagnostizierten Symptomen.

Die physiologische Voraussetzung für einen sicheren Gang ist ein ungestörtes Zusammenspiel des afferenten sensiblen Systems, der motorischen Impulse sowie der zentralmotorischen Steuerung und Koordination. Darüber hinaus bedarf es eines unbeeinträchtigten knöchernen und weichteiligen Bewegungsapparates [4].

Das afferente System liefert über sensible und sensorische Rezeptoren die notwendigen Informationen über die Lage des Körpers im Raum sowie die Stellung der Extremitäten und Gelenke an das zentrale Nervensystem. Dieses geschieht überwiegend durch die Propriozeption, die Lage- und Stellungsrezeptoren im Bereich der Sehnen und Gelenke sowie die Rückmeldungen des visuellen Systems und des Gleichgewichtsorgans im Innenohr. Das efferente motorische System zur Durchführung von Bewegungen besteht aus den absteigenden zentralen Nervenbahnen, den peripheren Nerven, der Muskulatur sowie den speziellen Strukturen der neuromuskulären Übertragung. Die Verarbeitung der afferenten Informationen und die Planung sowie das Intentionieren von Bewegungen erfolgt in verschiedenen Bereichen des zentralen Nervensystems. Hier sind speziell der Hirnstamm, das Kleinhirn, das extrapyramidale System der Basalganglien sowie der für die exekutive Bewegungssteuerung wesentliche präfrontale Cortex des Großhirns zu nennen. Jeder dieser Strukturen des zentralen oder peripheren Nervensystems können spezifische Symptome einer Funktionsstörung zugeordnet werden. Im Umkehrschluss kann aus jeder festgestellten Bewegungsstörung nach sorgfältiger Untersuchung ein Rückschluss auf die erkrankte anatomische Struktur erfolgen.

Vorausschauend sei hier darauf hingewiesen, dass unabhängig von den bisher genannten Strukturen und Funktionen Folgendes gezeigt werden konnte: Es besteht ein enger Zusammenhang zwischen rein kognitiven Defiziten und einer Beeinträchtigung des Gangbildes und der Gangsicherheit. Sowohl Störungen des Konzentrationsvermögens, der Aufmerksamkeit als auch der oben genannten exekutiven Funktionen können die Gehfähigkeit und damit die Mobilität erheblich beeinträchtigen [5].

Für die Bewertung und die anatomische Zuordnung einer Gang- oder Bewegungsstörung lassen sich bereits aus einer standardisierten klinischen Untersuchung die wichtigsten Aspekte ableiten [6]. Nach Ausschluss skelettogen bedingter Ursachen durch Fehlhaltungen, knöcherne Bewegungseinschränkungen oder Schmerzen lassen sich im normalen Gangbild Schrittlänge, Schrittfrequenz und die Breite der Fußstellung beschreiben. Auch die Körperhaltung und deren Reaktion auf Lage- und Stellungswechsel ergeben spezifische Hinweise auf die Art der zugrunde liegenden Erkrankung [7]. Lähmungen fallen durch Fehlfunktionen unter Belastung auf, z. B. das Absinken der Hüfte bei einer Schwäche der Beckenmuskulatur (Trendelenburg-Gang). Im Blindgang fallen durch den Wegfall der visuellen Kompensation Störungen der anderen Afferenzen auf: unsystematische Unsicherheit bei Störungen des Lagesinns und systematische Gangabweichungen bei Störungen des Gleichgewichtsorgans. Haltungsanomalien oder -instabilitäten des Rumpfes weisen auf zentrale Störungen der Koordination hin.

Zur Epidemiologie von Störungen der Geh- und Stehfähigkeit konnte im Rahmen der Bruneck-Studie gezeigt werden, dass in der norditalienischen Region Gangstörungen bei jedem 10. Bewohner im Alter zwischen 60 und 69 Jahren auftraten und bei mehr als 60 % der über 80-Jährigen. Neurologische Erkrankungen stellten bei deutlich mehr als der Hälfte der Fälle die größte Gruppe dar [1]. In etwa 30 % der Fälle lagen 2 oder mehr neurologisch bedingte Funktionsstörungen vor. Störungen der sensiblen Afferenzen und Erkrankungen aus dem Komplex der Parkinson-Syndrome waren die häufigsten Diagnosen.

Die klinische Relevanz von Gangstörungen zeigt sich in einer epidemiologischen Untersuchung [8], nach der etwa ein Drittel der über 65-Jährigen mindestens einmal pro Jahr stürzt und davon die Hälfte mehrmals. Etwa jeder 6. Sturz führt dabei zu relevanten Verletzungen wie Frakturen oder Schädelhirntraumata. Damit sind Gangstörungen häufiger Ursache von Stürzen als akute Erkrankungen wie Kreislaufreaktionen, Schlaganfälle oder Epilepsie. Unfallverletzungen stellen die fünfthäufigste Todesursache des älteren Menschen dar [9].

### Neurologische Erkrankungen mit Auswirkungen auf die Motorik

Nachfolgend werden relevante neurologische Krankheitsbilder dargestellt, welche speziell die Motorik und Auswirkungen auf die Gangsicherheit betreffen.

#### Lähmungen einzelner Muskeln und Muskelgruppen.

Durch die Schädigungen bzw. Erkrankungen einzelner Nerven, Muskeln oder Muskelgruppen kommt es zu umschriebenen Funktionsstörungen der Motorik. Typische Beispiele sind Druckschädigungen des Peronäusnervs oder die Kompression von Nervenwurzeln in der Lendenwirbelsäule. Beides kann zur Lähmung der Fußhebung mit der Gefahr von Stürzen durch unbemerktes Hängenbleiben mit der Fußspitze führen. Degenerative Muskelerkrankungen und Muskeldystrophien befallen bevorzugt die Hüft- und Beckenmuskulatur. Hierdurch werden die Rumpfstabilität und die Haltungskontrolle relevant beeinträchtigt. Sollte es keine kausale Behandlungsmöglichkeit geben, können hier orthopädische Maßnahmen, wie z. B. Schienen- und Orthesenversorgung, helfen.

#### Lumbalkanalstenose.

Eine typische Erkrankung des älteren Menschen. Ursächlich ist eine Einengung des Nervenkanals im Bereich der Lendenwirbelsäule mit einer Bedrängung der zum Bein ziehenden Nervenfasern [10]. Da diese Kompression statisch bedingt im Stehen deutlicher ausgeprägt ist als im Sitzen, treten Lähmungen der Beinmuskulatur und damit eine Beeinträchtigung der Gang- und Standsicherheit erst nach längerem Gehen auf und bilden sich im Sitzen und Liegen rasch zurück (Claudicatio spinalis). Behandlungsmöglichkeiten ergeben sich durch eine operative Dekompression.

#### Rückenmarksschädigung.

Ursächlich sind hierbei Erkrankungen oder Schädigungen im Spinalkanal oberhalb der Lendenwirbelsäule. Die häufigste Störung beim älteren Menschen ist eine Kompression im Bereich der Halswirbelsäule durch degenerative Veränderungen am Skelett- und Bandapparat (zervikale Myelopathie; [11]). Das resultierende spastische Gangbild ist durch eine Steifigkeit der Beine und unsicheres Aufsetzen geprägt. Da häufig auch die aufsteigenden Bahnen des Rückenmarks betroffen sind, wird die Gangsicherheit durch eine Verminderung der sensiblen Rückmeldung zusätzlich reduziert. Therapeutisch kommt bei funktionell relevanter Beeinträchtigung eine operative Dekompression infrage.

#### Hemiparese.

Die häufigste Ursache für eine Hemiparese im Alter ist vor allem ein Schlaganfall im Bereich des Großhirns. Aber auch andere Erkrankungen dieser Region wie Blutungen oder Tumoren können zu gleichartigen klinischen Bildern führen. Typisch ist eine gleichzeitige Lähmung von Gesicht, Arm und Bein derselben Seite. Die Gangsicherheit ist vordergründig durch die spastische Lähmung des Beines beeinträchtigt. Durch die zusätzliche Funktionsstörung des Armes sind die Möglichkeiten der Kompensation und des Schutzes im Falle eines Sturzes beeinträchtigt. Wie bei den meisten Erkrankungen des Großhirns ist darüber hinaus regelmäßig von zusätzlichen kognitiven Beeinträchtigungen auszugehen, sodass sich das Bild einer multimodalen Beeinträchtigung der Mobilität ergibt.

#### Ataxien.

Hiermit werden Koordinations- und Gangstörungen bezeichnet, die durch fehlerhafte sensible oder sensorische Rückmeldungen oder durch Störungen der zentralen Informationsverarbeitung bedingt sind. Das motorische System ist dabei nicht direkt betroffen, die Kraft der einzelnen Muskelgruppen ist regelrecht. Als wesentliche Symptome können die sensible, die vestibuläre und die zerebelläre Ataxie unterschieden werden, die in den folgenden Absätzen näher erläutert werden.

#### Sensible Ataxie.

Ursächlich hierfür ist eine Störung der sensiblen Rückmeldung über die Stellung von Muskeln und Gelenken an die zentralnervöse Steuerung. Im Alter liegt die häufigste Ursache in einer multilokulären Schädigung peripherer Nerven, einer Neuropathie. Diese kann als eigenständige degenerative Erkrankung oder als Komplikation anderer Grundkrankheiten, z. B. Diabetes mellitus, auftreten. Das Gangbild wird langsam, kleinschrittig, breitbasig und unsicher. Unebenheiten werden schlecht wahrgenommen und können nur bedingt ausgeglichen werden. Da der gestörte Lagesinn durch visuelle Kontrolle teilweise kompensiert werden kann, kommt es bei Dunkelheit oder anderen Beeinträchtigungen des Sehens zu einer Verdeutlichung der Symptomatik. Wenn die Behandlung einer Grunderkrankung nicht möglich ist, kommt therapeutisch Physiotherapie und ggf. die Versorgung mit einer Gehhilfe infrage.

#### Vestibuläre Ataxie.

Das vestibuläre Gleichgewichtsorgan im Innenohr ist von entscheidender Bedeutung für die Orientierung im Raum. Sensorische Funktionsstörungen treten vermehrt im höheren Lebensalter auf [12]. Die Symptomatik besteht in Drehschwindel mit der Folge systematischer Gangabweichungen zu einer Seite. Wie auch bei Störungen der Sensibilität sind hier teilweise Kompensationen durch das visuelle System möglich. Auch bei dieser Form der Gangunsicherheit kommt es bei Beeinträchtigungen des Sehens zu einer Verschlechterung.

#### Zerebelläre Ataxie.

Ursächlich hierfür sind Funktionsstörungen des Kleinhirns. Diese treten im Alter durch degenerative Erkrankungen, aber auch durch entzündliche und toxische, z. B. alkoholbedingte Schädigungen auf [13]. Diese Erkrankungen haben eine Beeinträchtigung der zentralen Steuerung der Bewegungskoordination zur Folge. Das klinische Bild ist eine Gang- und Standunsicherheit die sich besonders auf gezielte Bewegungen und das Gleichgewichtsgefühl auswirkt. Leitsymptom ist der durch gezielte Bewegungen verstärkte Intentionstremor. Da es sich um eine Störung der zentralen Koordination handelt, ist bei dieser Ataxie eine Kompensation durch das visuelle System nicht möglich. Die therapeutischen Möglichkeiten sind sehr beschränkt.

#### Parkinson.

Bei der Parkinson-Krankheit handelt es sich um eine fortschreitende degenerative Erkrankung des zentralen Nervensystems [14]. Sie gehört mit einer Prävalenz von ca. 20/1000 zu den häufigsten neurologischen Krankheitsbildern und Ursachen für eine Einschränkung der Mobilität. Im Vordergrund der Symptomatik steht eine komplexe Störung der Motorik, die durch eine Kombination von Verlangsamung, Steifigkeit und einen in Ruhe verdeutlichten Tremor geprägt ist. Zusätzlich wird die Stand- und Gangunsicherheit durch Instabilität der Haltungskontrolle mit erheblicher Sturzneigung verstärkt. Der Verlauf der Erkrankung ist fortschreitend. Im Falle der degenerativen Form dieser Erkrankung lässt sich der Verlauf therapeutisch beeinflussen und die Progredienz zumindest verlangsamen. Bei einem großen Teil der Betroffenen bestehen zusätzlich relevante kognitive Defizite.

#### Frontale Ataxie.

Diese spezielle Form der Gangstörung, bedingt durch eine Funktionsstörung frontotemporaler Hirnareale, ist eine typische Alterskrankheit. Sie kann durch unterschiedliche Mechanismen der Großhirnschädigung verursacht werden. Oft ist sie Folge der Summation multipler kleiner vaskulärer Läsionen (vaskuläre Enzephalopathie; [15]). Auch Störungen der Liquorzirkulation können ein ähnliches klinisches Bild hervorrufen (Normaldruckhydrozephalus; [16]). Fortgeschrittene degenerative Erkrankungen wie die Alzheimer-Krankheit oder frontotemporale Demenzen können ebenfalls ursächlich sein. Typisch ist ein breitbeiniger, kleinschrittiger, schleifender Gang. Die betroffene Person hat Schwierigkeiten, das Gehen zu initiieren. Die Füße werden kaum angehoben (Bügeleisengang) und die Rumpfkontrolle bei Lagewechsel ist erheblich beeinträchtigt. Therapeutisch gibt es beim Normaldruckhydrozephalus operative Ansätze.

## Eingeschränkte Mobilität durch Beeinträchtigung der Fahreignung

Krankheiten aus dem Bereich der Neurologie beeinträchtigen die Mobilität nicht nur durch die oben genannten Beschränkungen der körperlichen Bewegungsmöglichkeiten. Funktionsstörungen im Bereich des Sensoriums, der Sensibilität, der Motorik und der Koordination haben auch eine erhebliche Auswirkung auf die Fähigkeit, ein Kraftfahrzeug sicher und verantwortungsbewusst zu bedienen und zu führen.^1^ Dieser Problematik kommt eine besondere Bedeutung zu, da Autofahren für die Betroffenen ein naheliegender Kompensationsmechanismus ist, um die Mobilität trotz krankheitsbedingter Einschränkungen aufrechtzuerhalten.

Unter den Beeinträchtigungen des Sensoriums haben das Seh- und das Hörvermögen in Hinblick auf die Fahreignung eine besondere Bedeutung. Sehstörungen sind nicht nur durch direkte Erkrankungen des Auges bedingt, sondern auch durch Beeinträchtigungen der zerebralen Verarbeitung der Seheindrücke, die sich in Form von Gesichtsfeldstörungen oder anderen Störungen der Wahrnehmung zeigt. Schädigungen des motorischen Systems machen sich durch Lähmungen einzelner Muskeln oder von Muskelgruppen mit entsprechenden Funktionsstörungen bei der Bedienung eines Fahrzeuges (z. B. Bremsen, Lenken, Schalten) bemerkbar. Auch Ausfälle der Sensibilität können, wie oben beschrieben, Ursache erheblicher Beeinträchtigungen sein, da durch fehlende Rückmeldung über die Stellung von Gelenken der gezielte Einsatz von Muskeln, z. B. die Bedienung von Pedalen, bei fehlender optischer Kontrolle beeinträchtigt ist.

Neben somatosensorischen Funktionen ist die kognitive Leistungsfähigkeit ein wichtiger Faktor für die Fahreignung. Sie kann insbesondere durch Erkrankungen des Großhirns beeinträchtigt sein [17]. Neben der Bewusstseinslage spielen auch die Qualitäten Orientierung, Konzentration, Aufmerksamkeit und Reaktionsfähigkeit eine wichtige Rolle. Vor dem Hintergrund der Fahreignung ist zusätzlich die Beurteilung der Stabilität dieser kognitiven Leistungen unter Dauerbelastung von Relevanz. Die Manifestation solcher Symptome zeigt sich häufig nicht kontinuierlich. Bei kognitiven Störungen, insbesondere wenn übergeordnete koordinierende Steuerungsfunktionen des Großhirns betroffen sind, ist mit unerwartet und anfallsartig auftretenden Beeinträchtigungen zu rechnen. Andere Erkrankungen zeigen in der Initialphase zwar nur milde Funktionsausfälle, verlaufen dann aber mit einer vorhersagbaren Progredienz. Diese wäre bei der Beurteilung zu erwartender Beeinträchtigungen mitzuberücksichtigen. Dementsprechend sind Nachuntersuchungen zu planen. Bei vielen kognitiven Störungen ist zusätzlich zu beachten, dass sie von den Betroffenen krankheitsbedingt oft nicht wahrgenommen werden können und damit eigenverantwortliche Einschränkungen in Hinblick auf die Teilnahme am motorisierten Straßenverkehr nicht erwartet werden können. Hieraus ergibt sich eine besondere ärztliche Aufklärungspflicht.

Die beschriebenen Symptome können von unterschiedlichen Erkrankungen des Nervensystems hervorgerufen werden. Für die verkehrsmedizinische Begutachtung sollten die Symptome und deren funktionelle Auswirkungen im Vordergrund der Beurteilung stehen. Die Art der Erkrankung ist dann eher für die neurologische Einschätzung der Prognose und die weitere Verlaufsdynamik von Bedeutung. In der Anlage 4 der Fahrerlaubnis-Verordnung (FeV) werden aus dem Spektrum neurologischer Erkrankungen 6 Gruppen mit speziellen Richtlinien zur gutachterlichen Bewertung herausgestellt [18]. Diese sind 1. Erkrankungen des Rückenmarks, 2. Erkrankungen der neuromuskulären Peripherie, 3. die Parkinson-Erkrankung, 4. kreislaufabhängige Störungen der Hirntätigkeit, 5. strukturelle Hirnschäden und 6. Epilepsie.

Erkrankungen der Krankheitsgruppen 1. und 2. sind einfach zu diagnostizieren und in ihrer Relevanz für die Fahreignung einzuschätzen. Eine Kompensation wird in der Regel durch technische Maßnahmen der Fahrzeugausstattung zu erreichen sein. Schwieriger ist die Einschätzung der Erkrankungen mit zusätzlicher Beeinträchtigung kognitiver Funktionen. Speziell bei der Parkinson-Erkrankung, den kreislaufabhängigen Störungen und der Epilepsie liegt eine deutliche Altersabhängigkeit der Manifestationswahrscheinlichkeit, d. h. der Inzidenz, vor. Insofern bedürfen diese Erkrankungen vor dem Hintergrund der vorliegenden Thematik der besonderen Betrachtung.

### Neurologische Erkrankungen mit Auswirkungen auf die Fahreignung

#### Parkinson.

Für die Bewertung der Fahreignung bei Parkinson ist zu beachten, dass bei dieser Erkrankung multimodal mehrere Systeme betroffen sind [19]. Am auffälligsten sind die oben ausgeführten motorischen Symptome mit einer im Vordergrund stehenden Bewegungsarmut, einer Steifigkeit der Muskulatur und einem oft deutlichen Ruhetremor. Dabei wird leicht übersehen, dass der größte Teil dieser Menschen zusätzlich kognitive Beeinträchtigungen hat, die die Fahreignung oft wesentlich stärker beeinträchtigen können als die vordergründige motorische Symptomatik.

Aufgrund ihres degenerativen Charakters ist die Parkinson-Erkrankung chronisch fortschreitend. Durch verschiedene medikamentöse und neuerdings auch neurochirurgische Behandlungsmöglichkeiten lässt sich der Verlauf oft günstig beeinflussen und verlangsamen, jedoch nicht zum Stillstand bringen. Zusätzlich ist zu beachten, dass auch die medikamentöse Behandlung verkehrsmedizinisch relevante Nebenwirkungen hervorrufen kann. Hier ist besonders die Tagesmüdigkeit mit plötzlichen Schlafattacken zu nennen [20]. Psychotische Zustände mit Impulskontrollstörungen können zu risikoreichem und rücksichtlosem Fahren führen [21].

Die Fahreignung sollte im Falle einer Parkinson-Erkrankung zumindest infrage gestellt und einer besonders aufmerksamen Einschätzung unterzogen werden. Aufgrund der Komplexität der Symptomatik und deren Interaktion mit der Behandlung erscheinen neben neuropsychologischen Tests praktische Fahrprüfungen und Rückmeldefahrten besonders sinnvoll. Aufgrund der Progredienz der Erkrankung sollten regelmäßige Reevaluationen in ein- bis 2‑jährigen Abständen erfolgen [19].

#### Kreislaufabhängige Störungen der Hirnfunktion.

Diese Störungen sind im Alter die häufigsten neurologischen Erkrankungen. Etwa 5 % der Bevölkerung über 70 Jahre sind von den chronischen Folgen eines Schlaganfalls betroffen. Neben der akut auftretenden und anhaltenden Symptomatik können sich Durchblutungsstörungen des Gehirns auch als chronisch fortschreitende Beeinträchtigung im Sinne einer Demenz manifestieren (vaskuläre Enzephalopathie). Andererseits bleibt eine transiente Symptomatik (TIA) im Sinne einer passageren zerebralen Funktionsstörung ohne strukturelle Schädigung und damit ohne anhaltendes Defizit.

Die Beurteilung kreislaufbedingter Hirnleistungsstörungen unter dem Aspekt der Fahreignung erfolgt nach einem angemessenen Rehabilitationsintervall zunächst anhand der ggf. verbliebenen funktionellen Defizite. Da es sich allerdings bei diesen Erkrankungen in der Regel um Folgen allgemeiner Risikofaktoren, wie z. B. Arteriosklerose oder Bluthochdruck, handelt, ist die Einschätzung eines Rezidivrisikos für die Beurteilung der zukünftigen Fahreignung von wesentlicher Bedeutung. Eine Fahreignung erscheint nach längerem Intervall nur bei belegbar niedrigem Rezidivrisiko vertretbar. Unter Berücksichtigung individueller Risikofaktoren wie Alter und Begleiterkrankungen (Bluthochdruck, Diabetes u. a.) haben verschiedene neurologische Fachgesellschaften ein Positionspapier zur „Fahreignung bei Hirngefäßerkrankungen“ herausgebracht, das als Anhaltspunkt für die Einschätzung des Rezidivrisikos dienen kann [22].

#### Epilepsie.

Mit einer Prävalenz von etwa 0,5 % ist die Epilepsie ebenfalls eine der häufigen neurologischen Erkrankungen. Auch hier ist die Manifestation mit fortschreitendem Alter erhöht. Der Grund hierfür liegt in strukturellen Veränderungen und Schädigungen des Gehirns, die dann Ursache einer epileptischen Reaktion des geschädigten Hirngewebes sein können [23]. Nicht jeder epileptische Anfall ist mit einer manifesten Epilepsieerkrankung gleichzusetzen. So können auch im gesunden Gehirn durch spezielle exogene Provokationssituationen oder Noxen wie Medikamente oder Drogen einzelne Anfälle ausgelöst werden, ohne dass die Erkrankung diagnostiziert werden kann. Solche provozierten Anfälle ohne erkennbare Disposition für eine Epilepsieerkrankung treten bei 5–10 % der Bevölkerung auf.

Die Symptomatik des epileptischen Anfalls entsteht durch eine sich unkontrolliert in der Hirnrinde ausbreitende Aktivität, die zur Funktionsstörung oder zum Funktionsausfall der betroffenen Hirnregion führt. Hierbei kann es sowohl zu einer generalisierten Störung als auch zu Teilfunktionsstörungen kommen. Während der generalisierte Anfall in der Regel zu einem Koma und unwillkürlicher Aktivität der gesamten Muskulatur führt, präsentieren sich die partiellen Anfälle mit umschriebenen Störungen. Diese können Lähmungen oder Krämpfe einzelner Muskelgruppen sein sowie umschriebene Sensibilitätsstörungen oder inadäquate sensorische Phänomene. Es entstehen gelegentliche plötzliche Orientierungs- und Wahrnehmungsstörungen, die wiederum Ursache komplexer Fehlhandlungen im Straßenverkehr seien können. Diese Phänomene werden von der betroffenen Person oft nicht wahrgenommen oder sind nicht beeinflussbar, sodass eine erhebliche Gefahr für schwere Fahrfehler entstehen kann.

Epilepsiekranke zeigen im Intervall zwischen den Anfällen in der Regel einen unauffälligen Befund. Die Frage der Fahreignung hat sich deshalb im Wesentlichen mit dem Risiko von Anfallsrezidiven zu beschäftigen. Hierfür ist sowohl die Möglichkeit der Vermeidung anfallsauslösender Situationen als auch die Effektivität der Behandlung zu bewerten. Die Therapie erfolgt in der Regel medikamentös und ist in ca. 70 % erfolgreich. Für das Wiedererlangen der Fahrerlaubnis ist in der Regel eine mindestens einjährige Anfallsfreiheit zu fordern [24]. Zusätzlich ist zu berücksichtigen, ob eine der Epilepsie zugrunde liegende Erkrankung einen eigenständigen Einfluss auf die Fahreignung haben könnte.

## Fazit

Zusammenfassend ist festzustellen, dass neurologische Erkrankungen nicht selten im höheren Lebensalter die Mobilität im häuslichen Alltag einschränken. Darüber hinaus können sie auch die Eignung zum Führen eines Kraftfahrzeugs beeinträchtigen. Derartige neurologische Ausfälle können dann u. U. die Ursache „rätselhafter Unfälle“, z. B. durch akutes Versagen im Rahmen eines epileptischen Anfalls oder einer akuten Durchblutungsstörung des Gehirns sein. Oft sind diese Defizite nicht vorhersehbar oder auch krankheitsbedingt in ihrer Bedeutung nicht einschätzbar. Da aber, speziell bei den Hirndurchblutungsstörungen, oft spezifische Risikofaktoren bestehen oder, wie bei der Parkinson-Erkrankung, der fortschreitende Verlauf der Defizite vorhersagbar ist, ergeben sich gute Ansatzpunkte für eine frühzeitige ärztliche Aufklärung und ggf. für die Empfehlung, die Fahreignung überprüfen zu lassen, oder auch klar auf eine aktuell fehlende Fahreignung hinzuweisen.

## Footnotes

1 Siehe auch „Begutachtungsleitlinien zur Kraftfahreignung“ der Bundesanstalt für Straßenwesen (BASt): https://www.bast.de/DE/Verkehrssicherheit/Fachthemen/U1-BLL/BLL_node.html; zugegriffen: 11.06.2024.
